# Physical, Static, and Kinetic Analysis of the Electrochemical Deposition Process for the Recovery of Heavy Metal from Industrial Wastewater

**DOI:** 10.1155/2023/2741586

**Published:** 2023-01-07

**Authors:** Ridha Hamdi, Amani Rached, Amal L. Al–Otaibi, Imen Massoudi, Shouq Alkorbi, Amor Saidi Ben Ali

**Affiliations:** ^1^Department of Physics, College of Science, Imam Abdulrahman Bin Faisal University, P.O. Box 1982, 31441 Dammam, Saudi Arabia; ^2^Basic and Applied Scientific Research Center, Imam Abdulrahman Bin Faisal University, P.O. Box 1982, 31441 Dammam, Saudi Arabia; ^3^Department of Chemistry, College of Science, Imam Abdulrahman Bin Faisal University, P.O. Box 1982, 31441 Dammam, Saudi Arabia

## Abstract

Through the electrodeposition technique, toxic metals in wastewater can be removed and deposited on a chosen substrate with excellent selectivity. In this work, we use this technique to extract lead cations from simulated wastewater by using fluorine-doped tin oxide (FTO) substrate at various temperatures. In situ tracking of lead nucleation at advanced stages has been achieved by chronoamperometry. According to the experimental results, the theoretical models developed to study the kinetic growth of lead deposits in 2D and 3D are in good agreement. Nucleation rate and growth rate constants, for example, were found to be strongly influenced by temperature. Cottrell's equation is used to calculate the diffusion coefficient. X-ray diffraction, scanning electron microscopy, and energy-dispersiveX-ray techniques were used to investigate and characterize the lead deposits. The reported results could provide insight into the optimization of electrodeposition processes for heavy metal recovery from wastewater and electronic wastes.

## 1. Introduction

**Figure 1:**
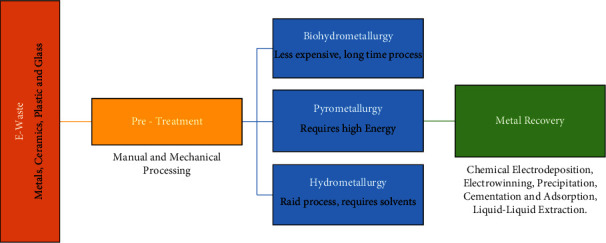
Diagram of metal recovery from e-waste from industrial water. In terms of pretreatment, there are two types: manual processing, which involves sorting, separating, cleaning, emptying, dismantling, decontaminating, and segregating, and mechanical processing, which involves shredding, milling, grinding, and separating through eddy current or air stream classifiers.

Treatment and recycling of wastewater and electronic waste (e-waste) have become increasingly necessary due to the presence of precious, critical, and strategic metals as well as the environmental impact of metal recovery [1–3]. e-waste is highly heterogeneous and includes a wide range of materials, such as metals, plastics, and ceramics, which are non-biodegradable pollutants. Mercury, lead, arsenic, cadmium, chromium, and cadmium, for example, are classified as the most common poisonous heavy metals, even at low levels of concentration [4, 5]. Both the U.S. Environmental Protection Agency and the International Agency for Cancer Research classify them as human carcinogens [6]. For instance, the levels of contamination in aquatic and terrestrial animals attributed to anthropogenic activities in Indonesia were found to have exceeded the tolerable limits of international standards, according to some reports [7–9]. Lead species are considered the most hazardous pollutants. It is extensively used in many different industries to fabricate, for instance, batteries, ammunition, metal products, and many other electronic devices. For example, in the United States in 2021, about 990,000 tons of secondary lead were produced from recycled materials, an amount equivalent to 62% of apparent domestic consumption. Nearly all the secondary lead was recovered from old scrap, mostly lead-acid batteries [10]. For these reasons, so far, numerous methods for recovering metal from e-waste have been suggested. Among them, one can cite cementation, ion exchange, liquid-liquid extraction (solvent extraction), and adsorption [11]. The major disadvantages of these methods are their slow kinetics, low adsorption capacity, and high cost. The precipitation of insoluble metal hydroxides is another used process [12]. New biochemical and biotechnological technologies are emerging. Even so, using these technologies poses a range of challenges [13]. Indeed, they involve chemical reactions that require the use of large quantities of expensive organic solvents and other polluting chemicals. Consequently, it is a priority to recover metals from e-waste via metallurgical extraction due to the fast depletion of natural mineral ores and the limited geographical availability of critical and/or strategic metals (Figure 1).

Therewith electrodeposition is an eco-friendly technology with convenient and precise controls as well as low energy consumption. It has been proven to be very effective in the treatment of e-waste [14]. It allows nanostructures to be deposited with a high degree of control [15, 16]. The electrodeposition process is either totally ohmic-regulated or mixed ohmic-diffusion controlled depending on the concentration of Pb (II) ions [17, 18]. Chen et al. [19] used a potentiostatic technique to depose Zn and Pb at varied temperatures and potentials into Cu foil. As known, the electrode structure could alter the electroplated deposits by modifying their nucleation energy, size, and shape. Indeed, FTO substrate is a widely used material for metal recovery due to its inert surface. It can be used to study the effects of metal-metal interaction on the growth and nucleation process [20, 21]. Lead electrodeposition onto a fluorine-doped tin oxide (FTO) substrate from a nitrate solution was investigated by Rebey et al. [22]. To the best of our knowledge, the coupling between the dynamics of lead recovery and the characterization of its deposits in FTO as a function of temperature has not yet been fully investigated.

This work intends to control the metal deposits on an FTO substrate and comprehend the processes that occur at the metal/substrate interfaces at different temperatures using an electrochemical cell connected to a chiller. Depending on the inquiry, either in situ chronoamperometry or cyclic voltammetry (CV) mode is used. Following that, we focus on how the 2D and 3D kinetic growth models relate to the experimental results and the theoretical development of lead deposits. X-ray diffraction (XRD), scanning electron microscopy (SEM), and energy-dispersiveX-ray (EDX) techniques were used to characterize the Pb deposits.

## 2. Materials and Methods

### 2.1. Materials

In deionized distilled water, the electrolyte solution, which is sodium nitrate (NaNO_3_) (Thermo Fisher Scientific, USA, 99.99%), was dissolved until a homogeneous, colorless, transparent liquid was obtained. High purity lead (II) nitrate (Pb(NO_3_)_2_) (Thermo Fisher Scientific, USA, 99.99%) was used as a Pb^2+^ ionic precursor. The obtained solution was continuously stirred until the complete dissolution of the chemicals. The electrical recovery process was carried out using a glass substrate made from FTO conducting glass of resistance 20 ± 0.3 Ω/square, and the glass is 2.2 mm thick with dimensions of 25 mm × 25 mm (Ossila Ltd, UK). The substrates were de-ionized after being washed with isopropyl alcohol to get them ready for experimentation. In order to complete the cleaning procedure, the substrates were heated in an electronic furnace at 80°C for 10 minutes. We run the electrochemical deposition experiments in a three-electrode cell in which three different electrodes (the working, counter, and reference) are placed in the same electrolyte solution. A platinum wire is used as the counter electrode, and the substrate is used as the working electrode. An Ag/AgCl electrode is used as the reference electrode.

### 2.2. Methods

#### 2.2.1. Voltammetry and Chronoamperometry

The cyclic voltammetric I–V and chronoamperometric measurements were carried out at varying temperatures (5°C, 20°C, and 35°C) using a HEKA potentiostat/galvanostat PG510 controlled by POSTMASTER software, at a scan rate of 60 mV/s. The electrochemical experiments were performed in an aqueous solution of 0.1 M Pb(NO_3_)_2_ in 0.4 M NaNO_3_. Cyclic voltammograms were measured in the voltage interval [–1.5 V, +1.5 V], while the chronoamperograms were recorded at the potential of –0.8 V.

#### 2.2.2. Characterisation

The structure and phase identification of the recovered electrodeposits were investigated using the following: (i) An X-ray diffraction analysis device (PanAlytical MPDPRO diffractometer) equipped with CuK*α* radiation of 1.540 Å over the range 20°–80° equipped with a linear X'Celerator detector using copper anticathode (*λKα*1/*α*2 = 1.540560/1.544330 Å). X-ray diffraction patterns are obtained in the 2*θ* range of 20–80°; the step size is 0.02; and the time per step is 30 s. (ii) A scanning electron microscope (SEM), an FEI Quanta 600 microscope and (iii) energy-dispersiveX-ray (EDX) coupled with the SEM were used.

## 3. Results and Discussion

### 3.1. Voltammetric and Chronoamperometric Behavior

**Figure 2:**
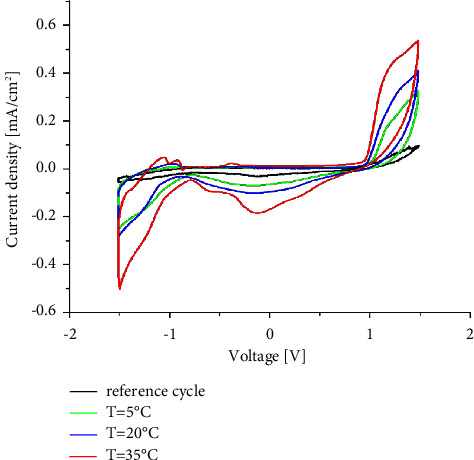
Typical cyclic voltammograms measured for the FTO electrodes over the same voltage range and different temperatures in pure distilled water, and the curves are obtained from an aqueous solution of 0.1 M Pb(NO_3_)_2_ in 0.4 M NaNO_3_.

The static behavior of lead electrosorption onto an FTO substrate is shown by the cyclic voltammograms recorded for the different temperatures of 5, 20, and 35°C (respectively, shown by the green, blue, and red lines in Figure 2). As shown, the reference I–V cycle without Pb^2+^ (black line) has a symmetrical shape and does not show any current peak. However, in the presence of lead, the current intensity measured through the voltage window seems somewhat sensitive to Pb^2+^ cations. There are clearly identifiable current density peaks at the potential of –0.8 V for all the studied samples at different temperatures. As the temperature increases, the peak intensity increases as well. These peaks indicate that electrochemical processes are successfully occurring on the electrode surface through the reduction of Pb^2+^ cations to Pb, with a strong dependence on the temperature parameter. As demonstrated later, the higher the temperature of the cell, the greater the density of the deposit of Pb [23, 24].

**Figure 3:**
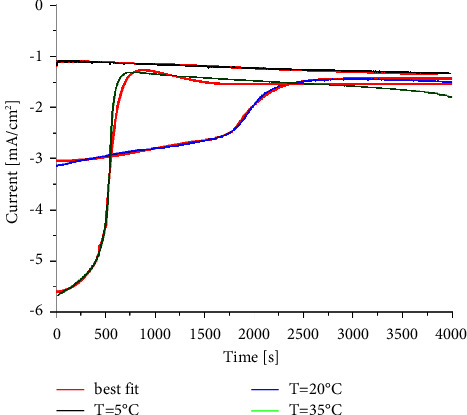
Current density toward time recorded during the electrodeposition of Pb^2+^ on an FTO electrode. Black, blue, and green lines are the experimental results for *T* = 5, 20, and 35°C, respectively. The red line corresponds to their best fit.

The in-situ evolution of the current transient is shown in Figure 3. They demonstrate that the nucleation rate changes with temperature. As shown, three distinct parts describe the whole process. Each current density reaches its threshold noted as *j*_*m*_ (maximum current density) at a time *t*_*m*_. Following that, they remain constant or decrease slightly. The first stage is characterized by a relatively stable current intensity, noted as the silent part. The duration of this stability is strongly affected by the temperature. Indeed, it decreases as the temperature increases. As the current density rises significantly, it enters the second stage of the nucleation process. As the temperature increases, the slope of the current density toward time increases clearly. During the third stage, the current density is practically stable. During this plateau-like shape phase, the Pb deposits across the FTO surface reach their equilibrium. As reported by González-García and his coworkers [25–27], electrochemical deposition is a very complex process and is strongly influenced by experimental conditions. Hence, these experimental results should be interpreted in light of theoretical, structural, and microstructural considerations.

### 3.2. Theoretical Approach

The elaboration of the crystalline materials involves phenomena of nucleation and growth. Electro-crystallization is the study of these two phenomena under the influence of an electric field. According to Amblard [28], these two phenomena compete with each other. They influence the kinetics, structure, and properties of the deposits. For example, the faster the nucleation speed, the finer the grains that form the deposit. Theoretical electrocrystallization models have been proposed to demonstrate the nucleation and growth modes during the electrodeposition process. The nucleation process is generally described in two types: instantaneous and progressive processes. The growth process is typically divided into three categories [29]: (i) The two-dimensional (2D) growth mode, or Frank–Van der Merwe mechanism, is generally found in the case where the metal and the substrate are of the same chemical nature. (ii) The 3D growth mode, or Volmer–Weber mechanism, can be exploited to produce nanostructures. (iii) Stranski–Krastanov's mechanism begins with a 2D growth mode followed by 3D growth. According to Stackelberg [30]:

(i) In the 2D growth process, the current density can be expressed by the following equations for instantaneous and progressive nucleation:
  (1) $$\begin{array}{c} j_{2D}^{inst}=\frac{zF\pi hMN_{0}k_{2D}^{2}t}{\rho }\exp\;\left(-\frac{\pi M^{2}N_{0}k_{2D}^{2}t^{2}}{\rho^{2}}\right)\text{,} \end{array}$$
  (2) $$\begin{array}{c} j_{2D}^{prog}=\frac{zF\pi hMA_{2D}k_{2D}^{2}t^{2}}{\rho }\exp\;\left(-\frac{\pi M^{2}A_{2D}k_{2D}^{2}t^{3}}{3\rho^{2}}\right)\text{,} \end{array}$$
  where *j*_2*D*_ is the current density, *k*_2D_ is the lateral growth rate constants (mol cm^−2^ s^–l^), *z*=+2 is the Pb valency, *F* is the Faraday constant (*F*=96485 C.mol^−1^), *t* is the time, *h* is the layer height (cm), *N*_0_ is the total number of active centers (cm^−2^), *A*_2D_ is the nucleation rate (nuclei cm^−2^ s^−1^), *M* is the atomic weight (g.mol^−1^), and *ρ* is the density (g.cm^−3^) of the deposit.
(ii) In the 3D growth process, the current density can be expressed by the following equations for instantaneous and progressive nucleation:
  (3) $$\begin{array}{c} j_{3D}^{inst}=zFk^{\prime }\left[1-\exp\;\left(-\frac{\pi M^{2}k^{2}N_{0}t^{2}}{\rho^{2}}\right)\right]\text{,} \end{array}$$
  (4) $$\begin{array}{c} j_{3D}^{prog}=zFk^{\prime }\left[1-\exp\;\left(-\frac{\pi M^{2}k^{2}A_{3D}t^{3}}{3\rho^{2}}\right)\right]\text{,} \end{array}$$
  where *k* and *k*′ are, respectively, the lateral and vertical growth rate constants and *A*_3D_ is the nucleation rate.
(ii) In the Stranski–Krastanov mechanism, the current density can be expressed through a combination of equations (1) or (2) and (3) or (4).

Furthermore, in Harrison and Thirsk's [31] studies, the current density is divided into two parts: the first one is due to 3D crystal growth, which can be given in equations (3) or (4). The second part is the current caused by an outward growth on a substrate base plane at a free surface uncovered by growing nuclei, *j*_*f*_. *j*_*f*_ is expressed by equations (5) or (6) for instantaneous and progressive nucleation, respectively, as follows:

(5) $$\begin{array}{c} j_{f}^{inst}=zFk_{0}\exp\;\left(-\frac{\pi M^{2}k^{2}N_{0}t^{2}}{\rho^{2}}\right)\text{,} \end{array}$$

(6) $$\begin{array}{c} j_{f}^{prog}=zFk_{0}\exp\;\left(-\frac{\pi M^{2}k^{2}A_{3D}t^{3}}{3\rho^{2}}\right)\text{,} \end{array}$$

where *k*_0_ is the growth rate constant on the base plane of the substrate. As shown in Figure 3, temperature influences the shape of current-time transients. As a result, temperature strongly affects the nucleation and growth processes. The behaviour of current-time characteristics is theoretically analysed at three temperatures (5°C, 20°C, and 35°C). Experimental results have been simulated and examined with 2D and 3D models according to equations (1) to (6). For the current density measured at 5°C (black curve), the fitting is described by the following equation, which is the combination of three parts:

(7) $$\begin{array}{c} J\left(t\right)=i_{0}\exp\;\left(-P_{1}t^{3}\right)+P_{2}t^{2}\;\exp\;\left(-P_{1}t^{3}\right)+P_{3}\left[1-\exp\;\left(-P_{4}{\left(t-t_{ind}\right)}^{3}\right)\right]\text{,} \end{array}$$

where

(8) $$\begin{array}{c} i_{0}=zFk_{0}\text{,}\\ P_{1}=\frac{\pi M^{2}A_{2D}k_{2D}^{2}}{3\rho^{2}}\text{,}\\ P_{2}=\frac{zF\pi hMA_{2D}k_{2D}^{2}}{\rho }\text{,}\\ P_{3}=zFk^{\prime }\text{,}\\ P_{4}=\frac{\pi M^{2}k^{2}A_{3D}}{3\rho^{2}}\text{,} \end{array}$$

where *t*_ind_ is the induction time.

The closest fit confirms that nucleation and crystal growth at 5°C begin with progressive nucleation and 2D crystal growth. At an induction time, *t*_0_, the second process of progressive nucleation and 3D crystal growth starts, as described by Stranski–Krastanov. The chronoamperometric curve of the electrodeposition of lead, obtained at 20°C (blue curve in Figure 3), shows that two quasi-plateaus appear at shorter and longer times, which follows the model of 3D growth [32]. To obtain further information regarding this process, different equations (from equations (1) to (6)) were tested. The most accurate fit of the experimental data is obtained for equation (9), derived from equations (3)–(5). Generally, this equation (9) indicates that after an induction time, there is instantaneous nucleation followed by progressive 3D nucleation:

(9) $$\begin{array}{c} J\left(t\right)=i_{0}\exp\;\left(-P_{1}^{\prime }t^{2}\right)+P_{3}\left[1-\exp\;\left(-P_{1}^{\prime }t^{2}\right)\right]+P_{3}^{\prime }\left[1-\exp\;\left(-P_{4}^{\prime }{\left(t-t_{ind}\right)}^{3}\right)\right]\text{,} \end{array}$$

where

(10) $$\begin{array}{c} P_{1}^{\prime }=\frac{\pi M^{2}k^{2}N_{0}}{\rho^{2}}\text{,}\\ i_{0}=zFk_{0}\text{,}\\ P_{3}=zFk^{\prime }\text{,}\\ P_{3}^{\prime }=zFk_{s}^{\prime }\text{,}\\ P_{4}^{\prime }=\frac{\pi M^{2}k_{s}^{2}A_{3D}}{3\rho^{2}}\text{,} \end{array}$$

where *k*_*s*_ and *k*_*s*_′ are, respectively, the lateral and vertical growth rate constants for the secondary growth process. At 35°C, the current-time transient curve has a steeper slope (the green curve in Figure 3). This mechanism can be described by progressive nucleation and 3D growth or the Volmer–Weber model [29]. The best fit can be achieved with the following equation, which is a combination of equations (4) and (6):

(11) $$\begin{array}{c} J\left(t\right)=i_{0}\exp\;\left(-P_{4}t^{3}\right)+P_{3}\left[1-\exp\;\left(-P_{4}t^{3}\right)\right]\text{.} \end{array}$$

**Figure 4:**
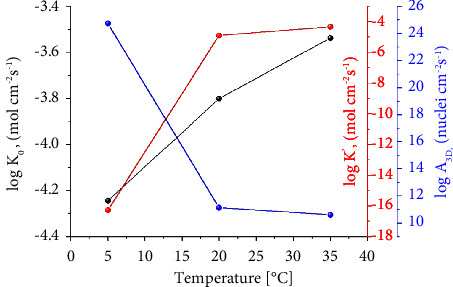
Dependence of log(*k*_0_), log(*k*′), and 3D on temperature for lead (II) on FTO.

Figure 4 shows the values of log   (*k*_0_), log   {*k*′), and *A*_3*D*_ as functions of temperature. They are derived from the best-fitting model. From 5°C to 35°C, the rate constant, *k*_0_, exhibits linear dependence on temperature (black curve). The vertical growth rate constant, *k*′, is found to be nonlinear for the first 3D growth process (red curve). Indeed, *k*′ increases as the temperature increases from 5°C to 20°C and then becomes practically stable from 20°C to 35°C. We also investigate the nucleation rate, *A*_3D_, assuming that the growth rates are the same in both directions. Our study shows that it decreases with increasing temperature, based on the blue curve.

### 3.3. Characterizations

**Figure 5:**
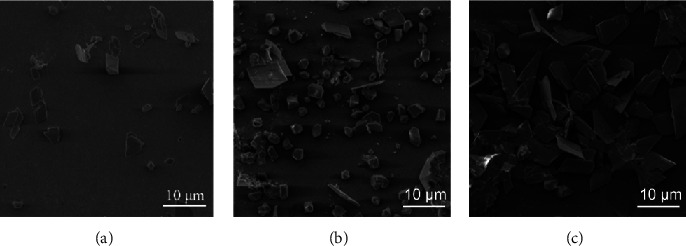
SEM micrographs of Pb deposits on FTO substrate at different temperatures. (a) *T* = 5°C, (b) *T* = 20°C, and (c) *T* = 35°C.

To correlate the chronoamperometry results, the morphologies of Pb deposit particles obtained at different temperatures are characterized by the SEM technique.  Figure 5 shows that the density of the individual crystals changes considerably with temperature, which enables the correlation of the lead (Pb) deposit morphology to their chronoamperograms. At 5°C, it is seen that randomly distributed individual crystals with sizes in the order of 5 *μ*m were formed on the substrate (Figure 5(a)). The crystals are characterized by flat faces and sharp corners and edges. At 35°C (Figure 5(c)). An agglomeration of crystals is observed. The distance between the deposits is lowered and one observes that sand-rose-like morphology develops at *T* = 20°C (Figure 5(b)). This can be explained by 3D crystal growth as described by the Stranski–Krastanov mechanism mentioned above. The obtained surface morphologies are in line with data obtained by chronoamperometry analysis [33]. Therefore, the nucleation mode of lead metal is strongly influenced by the concentration of cationic precursor, allowing the correlation of the morphology of the deposit with its CV. Previous studies attribute the shape of recovered metal deposits to the applied CV mode and deposition rate [32, 34]. Other physical factors such as the concentration of the ionic solution, the bathing temperature, and time are also taken into account to determine the shape and size of the recovered deposits. These include fernlike dendrites [35], needle-like [36], dendritic [32], and honeycomb-like structures [34].

**Figure 6:**
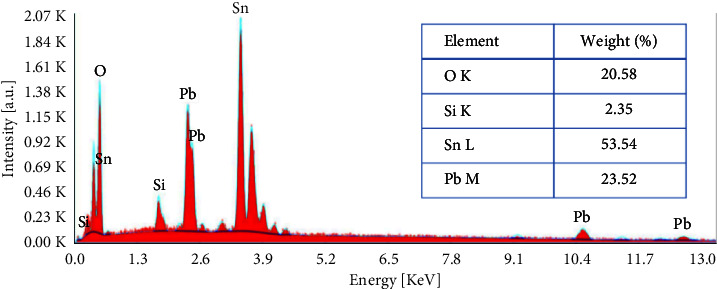
Energy-dispersiveX-ray analysis (EDX) spectrum of a lead deposit on an FTO substrate at 20°C.

Quantitative analysis of the obtained film is carried out using EDX. Results presented in Figure 6 show the presence of lead (Pb), oxygen (O), silicium (Si), and tin (Sn). Their percentage is given in the inset table. The results obtained from Figure 6 indicate Pb (23.52%), O (20.58%), Si (2.35%), and Sn (53.54%). Sn is the major constituent of the sample since it is the principal element of the substrate, Florine (F) doped SnO_2_. F is missed maybe due to its low doped percentage. Si is present in a low percentage because it is completely covered by the FTO substrate. The presence of Pb is the result of the electrodeposition process. Notably, we only provide the result for 20°C in Figure 6. For the sake of the figure's clarity, the other temperatures (5°C and 35°C) are not included because they behave in the same way.

**Figure 7:**
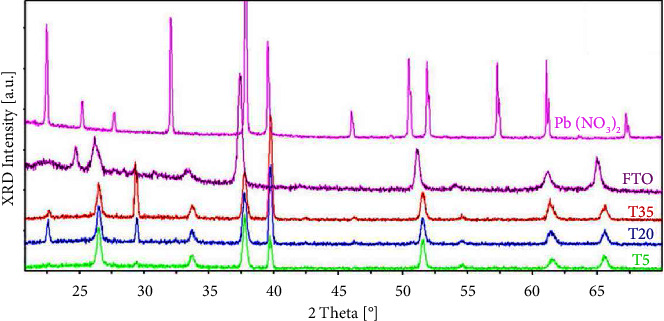
The XRD pattern of the lead deposited on an FTO substrate (with a potential pulse at −0.8 V at different temperatures). The patterns of Pb(NO_3_)_2_ and FTO are given for comparison.

The X-ray patterns of the obtained samples at 5°C (T5), 20°C (T20), and 35°C (T35) are compared to Pb(NO_3_)_2_ as starting materials and FTO as a substrate on which Pb is deposed (Figure 7). This figure shows the disappearance of lead nitrate in the three samples. Their patterns show peaks corresponding to FTO (SnO_2_: reference code 01–077–0452) in addition to extra lines observed at 22.5°, 29.5°, and 39.7°. No overlapping of the measured extra lines with those reported in the literature was found. Since the Pb (II) cations were electrochemically reduced at a potential of –0.8 V, the obtained deposit should be lead (lead oxide is excluded). A deep X-ray diffraction study is in progress to determine the structure of this new phase.

The crystallite size “*D*” is determined from XRD diffracted patterns through the Scherrer equations [36, 37]. The following equation is obtained:

(12) $$\begin{array}{c} D=\frac{K\lambda }{\beta \;\cos\;\theta }\text{ ,} \end{array}$$

where *K*=0.89 is a constant, *λ*=1.5406 Å, and *θ* and *β* are the diffraction angle and the corresponding full width at half-maximum of the observed peak, respectively. Lead is characterized mainly by two peaks observed at 2*θ*=26.2° and 2*θ*=39.7°. The size of particles is calculated from the first peak since the second corresponds to an overlapping of the (300) and (221) lines. For the three samples obtained at *T* = 5, 20, and 35°C, the calculated size is 45, 44, and 43 nm, respectively (Table 1). The crystallinity index (CI), which considers the contribution of the amorphous and crystalline phases, can be investigated by different methods, including XRD and NMR [36, 38, 39]. In this work, the crystallinity of deposited lead is calculated using X-ray diffraction data. The percentage of the CI of the sample toward temperature was determined for the entire diffracted pattern using the following formula:

(13) $$\begin{array}{c} CI\%=\frac{crystalline\;peak\;area}{noise}\times 100\text{.} \end{array}$$

**Figure 8:**
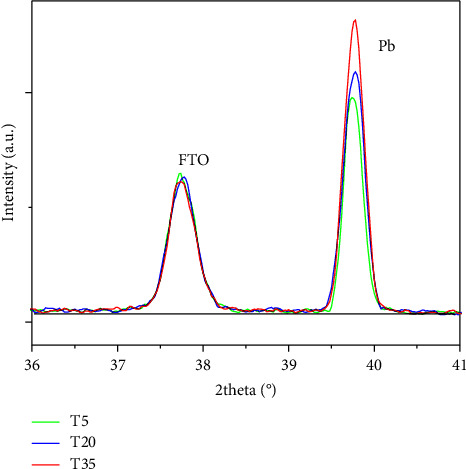
Characteristic peak positions of FTO and lead in the 2*θ* range 36° − 41° of samples obtained at 5, 20, and 35°C.

The obtained values are 67.3, 69.6, and 71.4% for *T* = 5°C, 20°C, and 35°C, respectively (Table 1). These values indicate an increase in crystallinity with temperature. Moreover, the increase of the diffracted peak related to lead (for example, the peak at 39.82°, Figure 8), as the temperature increases, is consistent with the SEM results (Figure 5), due to the agglomeration of the individual crystals.

## 4. Conclusion

Pb (II) cations were electrochemically reduced at a potential of −0.8 on the FTO substrate at different temperatures. The kinetic parameters of electrodeposition processes were determined from the theoretical analysis of chronoamperometry data. A reasonable agreement between the values of diffusion coefficients determined by applying the Cottrell equation and the nonlinear fitting method was achieved. The density number of active sites and the nucleation rate constant have been discussed. According to our results, the morphology of deposit particles and chronoamperometry curves are well correlated. After comparison with what was reported in the literature, XRD and EDX analyses suggest the formation of a novel lead phase. This study highlights the importance of controlling the recovery process of toxic e-waste from industrial water.

## Figures and Tables

**Table 1:** Crystallite size and crystallinity index for the samples obtained at 5°C (T5), 20°C (T20), and 35°C (T35).

	Crystallite size (nm)	Crystallinity index (%)
T5	45	67.3
T20	44	69.6
T35	43	71.4

## Data Availability

Data are available on request from the corresponding author.
